# Inhibition of autophagy and MEK promotes ferroptosis in Lkb1-deficient Kras-driven lung tumors

**DOI:** 10.1038/s41419-023-05592-8

**Published:** 2023-01-26

**Authors:** Vrushank Bhatt, Taijin Lan, Wenping Wang, Jerry Kong, Eduardo Cararo Lopes, Jianming Wang, Khoosheh Khayati, Akash Raju, Michael Rangel, Enrique Lopez, Zhixian Sherrie Hu, Xuefei Luo, Xiaoyang Su, Jyoti Malhotra, Wenwei Hu, Sharon R. Pine, Eileen White, Jessie Yanxiang Guo

**Affiliations:** 1 Rutgers Cancer Institute of New Jersey, New Brunswick, NJ 08901 USA; 2 Department of Medicine, Rutgers Robert Wood Johnson Medical School, New Brunswick, NJ 08901 USA; 3 Department of Pharmacology, Rutgers University, Piscataway, NJ 08903 USA; 4 Department of Molecular Biology and Biochemistry, Rutgers University, Piscataway, NJ 08854 USA; 5 Ludwig Princeton Branch, Ludwig Institute for Cancer Research, Princeton University, Princeton, NJ 08540 USA; 6 Department of Chemical Biology, Rutgers Ernest Mario School of Pharmacy, Piscataway, NJ 08854 USA

**Keywords:** Targeted therapies, Cancer metabolism, Non-small-cell lung cancer

## Abstract

LKB1 and KRAS are the third most frequent co-mutations detected in non-small cell lung cancer (NSCLC) and cause aggressive tumor growth. Unfortunately, treatment with RAS-RAF-MEK-ERK pathway inhibitors has minimal therapeutic efficacy in LKB1-mutant KRAS-driven NSCLC. Autophagy, an intracellular nutrient scavenging pathway, compensates for Lkb1 loss to support Kras-driven lung tumor growth. Here we preclinically evaluate the possibility of autophagy inhibition together with MEK inhibition as a treatment for Kras-driven lung tumors. We found that the combination of the autophagy inhibitor hydroxychloroquine (HCQ) and the MEK inhibitor Trametinib displays synergistic anti-proliferative activity in *Kras*^*G12D/+;*^*Lkb1*^*-/-*^ (KL) lung cancer cells, but not in *Kras*^*G12D/+;*^*p53*^*-/-*^ (KP) lung cancer cells. In vivo studies using tumor allografts, genetically engineered mouse models (GEMMs) and patient-derived xenografts (PDXs) showed anti-tumor activity of the combination of HCQ and Trametinib on KL but not KP tumors. We further found that the combination treatment significantly reduced mitochondrial membrane potential, basal respiration, and ATP production, while also increasing lipid peroxidation, indicative of ferroptosis, in KL tumor-derived cell lines (TDCLs) and KL tumors compared to treatment with single agents. Moreover, the reduced tumor growth by the combination treatment was rescued by ferroptosis inhibitor. Taken together, we demonstrate that autophagy upregulation in KL tumors causes resistance to Trametinib by inhibiting ferroptosis. Therefore, a combination of autophagy and MEK inhibition could be a novel therapeutic strategy to specifically treat NSCLC bearing co-mutations of LKB1 and KRAS.

## Introduction

Lung cancer is a leading contributor to cancer-attributed deaths in the United States, with an overall survival rate of 17% at any stage and 4% for more advanced stages [1]. About 85% of lung cancers are characterized as non-small cell lung carcinoma (NSCLC) [2]. KRAS mutations in NSCLC usually signify a poor prognosis and are linked with resistance to multiple cancer treatments [3]. Functional, predictive biomarkers play an essential role in predicting response to different treatments to enhance prognosis, thereby enabling the formulation of individualized therapies. Mutations in the epidermal growth factor receptor (EGFR), KRAS, Fibroblast growth factor receptor 1 (FGFR1), Anaplastic lymphoma kinase (ALK), MET, PIK3CABRAF, ROS1, NTRK, RET, HER2, LKB1, and TP53 [4–7] have been used as biomarkers to guide targeted cancer treatment. In particular, tumors with LKB1 mutations, which are detected in 15–30% of NSCLC, including tumors with co-occurring KRAS mutations detected in 5–10% of NSCLC [8, 9], unfortunately lack effective targeted therapies and are associated with the resistance to immunotherapy [6].

TP53 and LKB1 mutations in KRAS-mutant NSCLC show distinct genetic profiles and responses to therapies [6]. While co-mutations in KRAS and LKB1 are found in tumors of squamous cell carcinoma and adenocarcinoma, KRAS and TP53 co-mutations are found only in adenocarcinoma [10, 11]. Whereas Kras-p53 mutant tumors respond to combination therapy with docetaxel and the MEK inhibitor Selumetinib, Kras-Lkb1 mutant tumors do not [12]. Kras-p53 mouse lung tumors are also more responsive to radiotherapy compared to tumors harboring Kras-Lkb1 mutations [13, 14]. Therefore, there is an urgent need for precision therapy for different subgroups of KRAS-driven NSCLC.

Except for KRAS^G12C^ [15, 16], targeting RAS directly remains a challenge for NSCLC therapy [17]. Moreover, inhibiting downstream effectors of RAS signaling such as the MAP kinase pathway has not produced durable responses [18–20]. The contemporary strategies for lung cancer therapy are centered on the rationale of combining several different treatment modalities that produce the best patient outcomes [21]. Treatment with MEK inhibitors often forms the basis for lung cancer combination therapies [22, 23]. Trametinib, a MEK1/2 inhibitor, was the first agent approved by the FDA in 2013 for the treatment of BRAF V600E-mutant metastatic melanoma, followed by Cobimetinib [23, 24]. A combination of Dabrafenib and Trametinib was approved by the FDA in 2017 for the treatment of metastatic NSCLC harboring BRAF V600E-mutations [24]. The use of Trametinib as a single agent has been shown to induce resistance via overactivation of BRAF or PI3K/Akt pathways [25] and activation of the IGF1R–MEK5–Erk5 pathway [26]. In addition, the adverse side effects of MEK inhibitors [27] prompt the urgent need to develop other novel combination strategies to reduce drug-induced toxicities and suppress conventional therapy-induced resistance.

Autophagy -a lysosomal-mediated cannibalization process- captures, degrades, and recycles subcellular macromolecules and organelles [28]. Using genetically engineered mouse models (GEMMs) to conditionally delete essential autophagy genes, our group and others have demonstrated that autophagy is upregulated and promotes tumorigenesis in different types of cancer by multiple tumor cell-autonomous mechanisms [29–37]. Besides, autophagy counteracts oncogenic stress to support the malignant transformation transition driven by mutated RAS [38]. Autophagy also supports tumorigenesis by tumor cell non-autonomous mechanisms, including promotion by systemic autophagy through the maintenance of circulating arginine [37, 39], enabling secretion of alanine and other amino acids into the tumor microenvironment by stromal-associated pancreatic stellate cells [36, 40], and suppression of anti-tumor T-cell responses through autophagy-mediated “hepatic autophagy immune tolerance” [37, 41]. We have previously reported that autophagy inhibition during *Kras*^*G12D/+;*^*Lkb1*^*-/-*^ (KL) lung tumorigenesis is synthetically lethal [5]. Lack of autophagy contributes to metabolic dysfunction of KL lung tumors, which is caused by reduced levels of amino acids available to sustain mitochondria energy generation in starved cancer cells. This further leads to autophagy-deficient cancer cells to extract energy via fatty acid oxidation (FAO), resulting in a decreased lipid reserve and subsequent energy crisis [5]. Recently, using an autophagy loss switchable GEMMs for *Kras*^*G12D/+;*^*p53*^*-/-*^ (KP) NSCLC, we demonstrated that transient systemic autophagy ablation is selectively and irreversibly deleterious to lung cancer by impairing lung tumor cell metabolism and promoting T-cell mediated tumor killing [37]. Thus, autophagy promotes cancer by enhancing metabolic fitness and limiting immune and non-immune tumor suppression mechanisms, providing strong evidence that targeting the autophagy pathway with small molecule inhibitors would be an effective novel therapeutic strategy.

In this study, we demonstrated that inhibition of the RAS -> MEK -> ERK pathway induced lung cancer cells to use autophagy as a survival mechanism. Importantly, combining the autophagy inhibitor hydroxychloroquine (HCQ) together with the MEK inhibitor Trametinib elicited a synergistic anti-proliferative effect in KL mouse lung tumor-derived cell lines (TDCLs), and led to tumor regression in vivo. However, such an effect was not observed in KP TDCLs nor in in vivo KP tumor models. Compared with the single agents, the combination therapy significantly reduced basal mitochondrial respiration and ATP production, with this effect limited only to KL lung cancer cells. We further identified that several glycolytic and TCA cycle metabolites upregulated in KL lung tumors of mice by Trametinib were significantly reduced by the combination treatment. Consistent with the altered levels of glycolytic and TCA cycle metabolites, the carbon flux from glucose to the intermediates of glycolysis and TCA cycle in KL lung tumors was significantly reduced by the combination treatment compared with the vehicle control. Finally, we demonstrated that the combination of HCQ and Trametinib induced ferroptosis in KL cancer cells, leading to tumor regression. Thus, a combination of autophagy and MEK inhibition could be a novel therapeutic strategy to specifically treat NSCLC harboring co-mutations of KRAS and LKB1.

## Results

### Autophagy inhibition sensitized KL lung cancer cells to the MEK inhibitor Trametinib

**Fig. 1:**
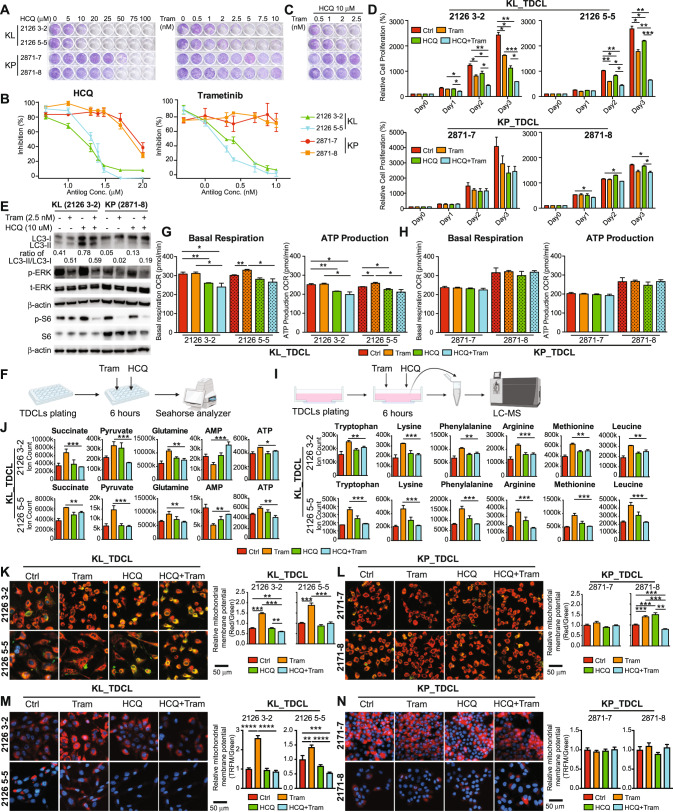
Inhibition of autophagy by HCQ resulted in KL TDCL, but not KP TDCL, to be sensitive to MEK inhibitor Trametinib. **A** Clonogenic survival assay of KL (clone 2126 3-2 and clone 2126 5-5) and KP (clone 2871-7 and clone 2871-8) TDCLs treated with HCQ or Trametinib individually at indicated concentrations. **B** Cell growth inhibition curve of KL (clone 2126 3-2 and clone 2126 5-5) and KP (clone 2871-7 and clone 2871-8) TDCLs treated with HCQ or Trametinib individually at indicated concentrations in Table 1. **C** Clonogenic survival assay of KL (clone 2126 3-2 and clone 2126 5-5) and KP (clone 2871-7 and clone 2871-8) TDCLs treated with the combination of HCQ and Trametinib at indicated concentrations. **D** Relative proliferation of KL (clone 2126 3-2 and clone 2126 5-5) and KP (clone 2871-7 and clone 2871-8) TDCLs treated with vehicle control, HCQ (10 μM), Trametinib (2.5 nM) and the combination. **E** Western blot for LC3, pERK, total ERK, pS6, total S6 and β-actin of KL and KP TDCLs treated with vehicle control, HCQ (10 μM), Trametinib (2.5 nM) and the combination for 6 h. **F** Scheme of the KL or KP TDCLs for measuring oxygen consumption rate (OCR) using Seahorse XFe24 analyzer. **G** Basal respiration and ATP production of KL TDCLs (clone 2126 3-2 and clone 2126 5-5 (with black squares)) after 6 h’ treatment with vehicle control, HCQ (10 μM), Trametinib (2.5 nM) and the combination. **H** Basal respiration and ATP production of KP TDCLs (clone 2871-1 and clone 2871-8 (with black squares)) after 6 h’ treatment with vehicle control, HCQ (10 μM), Trametinib (2.5 nM) and the combination. **I** Scheme of the metabolomics analysis via LC-MS of KL TDCLs after 6 h’ treatment. **J** The levels of metabolites of KL (clone 2126 3-2 and clone 2126 5-5) TDCLs after 6 h’ treatment with vehicle control, HCQ (10 μM), Trametinib (2.5 nM) and the combination. **K** Left: Overlapping images of KL (clone 2126 3-2 and clone 2126 5-5) TDCLs treated with vehicle control, HCQ (10 μM), Trametinib (2.5 nM) and the combination for 6 h and stained with MitoTracker Red CMXRos for mitochondrial membrane potential and MitoTracker Green FM for mitochondrial mass. Blue: Hoechst 33342 for nuclear staining. Right: graph of the relative mitochondrial membrane potential of KL TDCLs quantified by the ratio of red fluorescence intensity and green fluorescence intensity. **L** Left: Overlapping images of KP TDCLs (clone 2871-7 and clone 2871-8) treated with vehicle control, HCQ (10 μM), Trametinib (2.5 nM) and the combination for 6 h and stained with MitoTracker Red CMXRos for mitochondrial membrane potential and MitoTracker Green FM for mitochondria mass. Blue: Hoechst 33342 for nuclear staining. Right: graph of relative mitochondrial membrane potential of KP TDCLs quantified by the ratio of red fluorescence intensity and green fluorescence intensity. **M** Left: Overlapping images of KL (clone 2126 3-2 and clone 2126 5-5) TDCLs treated with vehicle control, HCQ (10 μM), Trametinib (2.5 nM) and the combination for 6 h and stained with TMRM (red fluorescence) for mitochondrial membrane potential. Blue: Hoechst 33342 for nuclear staining. Right: graph of the relative mitochondrial membrane potential of KL TDCLs quantified by the ratio of red fluorescence intensity and total cell numbers. **N** Left: Overlapping images of KP (clone 2871-1 and clone 2871-8) TDCLs treated with vehicle control, HCQ (10 μM), Trametinib (2.5 nM) and the combination for 6 h and stained with TMRM (red fluorescence) for mitochondrial membrane potential. Blue: Hoechst 33342 for nuclear staining. Right: graph of the relative mitochondrial membrane potential of KP TDCLs quantified by the ratio of red fluorescence intensity and total cell numbers. Data are mean ± s.e.m. *p < 0.05, **p < 0.01, ***p < 0.001, ****p < 0.0001.

MEK inhibitors have gained approval for treating patients with BRAF- and KRAS-driven NSCLC in combination with other standard therapies. However, acquired resistance to MEK inhibitors is still a major challenge [18–20]. RAS activation renders tumor cells addicted to autophagy, enabling survival during metabolic stress [42–44]. We previously found that autophagy activation compensates for the loss of Lkb1 in Kras-driven lung tumorigenesis [5], which prompted us to preclinically evaluate the potential of using autophagy inhibition together with FDA-approved MEK/ERK inhibitors for the treatment of KL NSCLC. While autophagy-specific inhibitors are currently under investigation, we treated KL and KP mouse lung TDCLs with the FDA-approved autophagy nonspecific inhibitor HCQ and Trametinib at increasing concentrations. Compared with KP TDCLs, KL TDCLs were more sensitive to HCQ- or Trametinib-induced cell growth arrest (Fig. 1A, B). Most importantly, the low concentration of single agents had minimal effect in causing cell growth arrest in KL TDCLs, but the combination treatment synergistically inhibited cell proliferation (Fig. 1C, D), which was also confirmed by the calculation of the combination index (Table 1). We did not observe the synergistic anti-proliferation effect with the same concentration of these reagents in KP TDCLs (Fig. 1C, D), although the inhibition of phosphorylation of ERK and S6, and the accumulation of LC3-II by the combination therapy were observed in both KL TDCLs and KP TDCLs after 6 h drug treatment (Fig. 1E, Supplemental Fig. 1). Our observations indicate that the combined inhibition of autophagy and MEK might be particularly effective in the treatment of KL lung cancer.

**Table 1:** DRUG COMBINATION in Vitro CALCUSYN REPORT (Fa-CI table with Fa increment of 0.05).

HCQ (uM)	Tram (nM)	Fa	CI
5	0.5	0.217568	0.334
5	1	0.276794	0.418
5	2	0.511382	0.526
5	2.5	0.559375	0.593
10	0.5	0.36757	0.497
10	1	0.576203	0.513
10	2	0.446177	0.739
10	2.5	0.440986	0.832
20	0.5	0.629069	0.75
20	1	0.782879	0.731
20	2	0.787708	0.868
20	2.5	0.912905	0.79
25	0.5	0.835889	0.762
25	1	0.892805	0.763
25	2	0.91607	0.848
25	2.5	0.93929	0.856
30	0.5	0.808339	0.931
30	1	0.927181	0.83
30	2	0.836887	1.101
30	2.5	0.975435	0.829

*Fa* fraction affected, *CI* Combination Index.

CI < 1, =1 and >1 indicates synergism, additive effect and antagonism, respectively.

At Fa>0.45 showed synergistic effect (CI < 1).

For anti-cancer agents, synergism (CI < 1) at high dose (high effect) is more relevant to the therapy than the CI values at low dose (low effect).

### Combination of HCQ and Trametinib impaired KL TDCL mitochondrial metabolism

Autophagy is essential to maintain mitochondrial metabolism for cancer cell survival during metabolic stress [5, 30, 32, 45]. To understand the mechanism of synergistic anti-proliferation by the combination treatment in KL TDCLs, KL and KP TDCLs were treated with the single agents or their combination for 6 h and the mitochondrial function was examined using the Seahorse XFe24 Analyzer (Fig. 1F). Combination treatment significantly inhibited basal respiration and ATP production compared with the vehicle control or Trametinib alone in KL TDCLs (Fig. 1G), but not in KP TDCLs (Fig. 1H). Further, metabolomics analysis (Fig. 1I) of TDCLs observed that treatment with Trametinib significantly increased the levels of several glycolytic and TCA cycle intermediates in KL TDCLs, which were reduced upon co-treatment with HCQ (Fig. 1J). In connection with these changes, Trametinib treatment suppressed AMP levels and increased ATP levels in KL cells, which was corrected by co-treatment with HCQ (Fig. 1J). The mitochondrial membrane potential generated by proton pumps is an essential component in the process of energy storage during oxidative phosphorylation. Together with the proton gradient, mitochondrial membrane potential forms the transmembrane potential of hydrogen ions which is harnessed to make ATP [46]. Hence, we measured the mitochondrial membrane potential using MitoTracker Red CMXRos and observed that Trametinib treatment alone significantly increased mitochondrial membrane potential compared to vehicle control in KL TDCLs, which was significantly dampened by the combination of HCQ and Trametinib (Fig. 1K), but not in KP TDCLs (Fig. 1L). Such reduced mitochondrial membrane potential by the combination treatment in KL TDCLs, not in KP TDCLs, was further validated using a standard less oxidative stress-sensitive dye TMRM (Fig. 1M, N). The combination treatment also reduced the levels of several amino acids that were upregulated upon treatment with Trametinib (Fig. 1J). Taken together, we found that the combination of HCQ and Trametinib in KL cells impairs functional mitochondria, leading to energy crises that could prevent cell proliferation and induce cell death.

### Combination of HCQ and Trametinib synergistically inhibited KL allograft tumor growth, but not KP allograft tumor growth

**Fig. 2:**
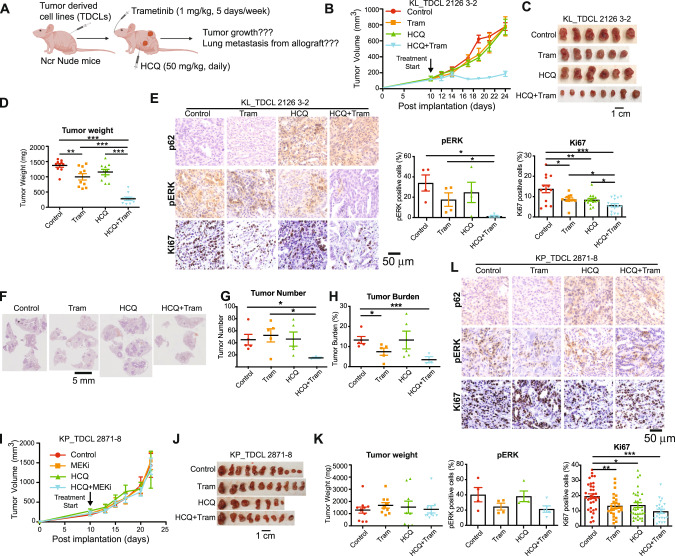
Combination of HCQ and Trametinib inhibited the growth of established KL allograft tumors, and not KP allograft tumors. **A** Scheme of an allograft mouse model in immunodeficient Ncr nude mice treated with vehicle control, HCQ (50 mg/kg, daily, I.P.), Trametinib (1 mg/kg, 5 days/week, oral gavage), or the combination. **B** Graph of KL allograft tumor growth in Ncr nude mice treated with vehicle control, HCQ, Trametinib, or the combination. **C** Gross pathology of KL allograft tumors in Ncr nude mice treated with vehicle control, HCQ, Trametinib or the combination. **D** Tumor weight of KL allograft tumors in Ncr nude mice treated with vehicle control, HCQ, Trametinib or the combination. **E** IHC for p62, pERK and Ki67 of KL allograft tumor from (**C**) (left panel) and quantification of pERK and Ki67 (right panel). **F** Representative H&E staining of lung tissues shows spontaneous lung metastasis from KL allograft tumors in Ncr nude mice. G&H. Quantification of tumor number (**G**) and tumor burden (**H**) from (**F**). **I** Graph of KP allograft tumor growth in Ncr nude mice treated with vehicle control, HCQ (50 mg/kg, daily, I.P.), Trametinib (1 mg/kg, 5 days/week, oral gavage), or the combination. **J** Gross pathology of KP allograft tumors in Ncr nude mice treated with vehicle control, HCQ, Trametinib or the combination. **K** Tumor weight of KP allograft tumors in Ncr nude mice treated with vehicle control HCQ, Trametinib or the combination. **L** IHC for p62, pERK and Ki67 of KP allograft tumor from (**J**) (top panel) and quantification of pERK and Ki67 (bottom panel). Data are mean ± s.e.m. *P < 0.05; **P < 0.01; ***P < 0.001.

To further validate our hypothesis that combining autophagy and MEK inhibitors could be a novel therapeutic strategy specifically in the treatment of KL NSCLC, we first assessed the anti-tumor effect of the combination therapy in established KL and KP TDCL-induced allograft tumors in immunodeficient NCr nude mice. When the allograft tumors were measurable, the mice were treated with HCQ or Trametinib alone, or with a combination of both (Fig. 2A). Although the single agents had no effect in reducing tumor growth, the combination treatment significantly reduced KL allograft tumor growth (Fig. 2B–D), which was accompanied by reduced cell proliferation (Ki67) and pErk in these tumors compared to the tumors collected from the mice treated with the single agents or vehicle control (Fig. 2E). Moreover, p62 accumulation, indicative of autophagy blockade, was observed in the tumors collected from the mice treated with HCQ or the combination (Fig. 2E). Compared to KP lung tumors, KL lung tumors have increased metastasis [11]. We observed that the combination treatment significantly inhibited spontaneous lung metastasis migrated from KL allograft tumors in NCr nude mice assessed by the quantification of tumor number and tumor burden of scanned lung sections (Fig. 2F–H). However, combination treatment did not cause any tumor growth inhibition of KP allograft tumors in NCr nude mice (Fig. 2I–L).

### Combination of HCQ and Trametinib synergistically inhibited established KL lung tumor growth, but not KP lung tumor growth

**Fig. 3:**
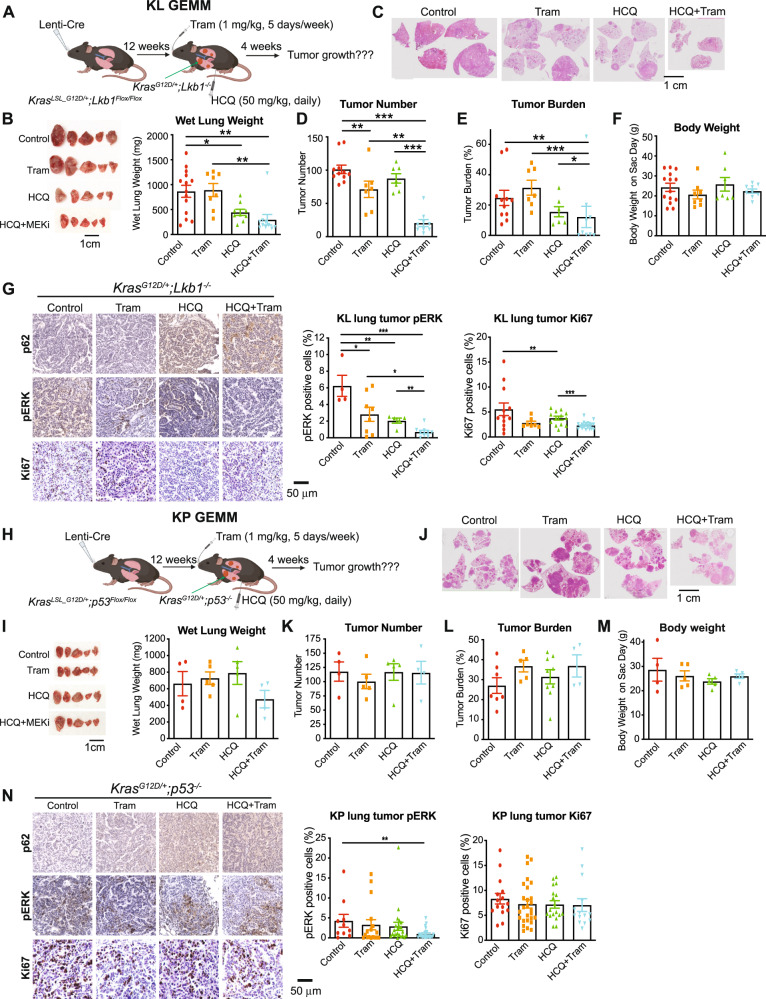
HCQ increased the sensitivity of KL lung tumors, and not KP lung tumors, to MEK inhibitor Trametinib. **A** Scheme of *Kras*^*LSL_G12D/+*^*;Lkb1*^*Flox/Flox*^ GEMM bearing KL lung tumors treated with vehicle control, HCQ (50 mg/kg, daily, I.P.), Trametinib (1 mg/kg, 5 days/week, oral gavage), or the combination. **B** Representative gross lung pathology (left panel) and wet lung weight (right panel) of mice bearing KL lung tumors treated with vehicle control, HCQ, Trametinib or the combination for four weeks. **C** Representative H&E staining of lung tissues of the mice from (**B**). **D**, **E** Quantification of tumor number (**D**) and tumor burden (**E**) from (**C**). **F** The body weight of the mice bearing KL lung tumor on the day of sacrifice. **G** IHC for p62, pERK, and Ki67 of KL lung tumors from (**B**) (left panel) and quantification of pERK and Ki67 (right panel). **H** Scheme of *Kras*^*LSL_G12D/+*^*;p53*^*Flox/Flox*^ GEMM bearing KP lung tumors treated with vehicle control, HCQ (50 mg/kg, daily, I.P.), Trametinib (1 mg/kg, 5 days/week, oral gavage), or the combination. **I** Representative gross lung pathology (left panel) and wet lung weight (right pane) of mice bearing KP lung tumors treated with vehicle control, HCQ, Trametinib or the combination for four weeks. **J** Representative H&E staining of lung tissues of the mice from (**I**). **K**, **L** Quantification of tumor number (**K**) and tumor burden (**L**) from (**J**). **M** The body weight of the mice bearing KP lung tumor on the day of sacrifice. **N** IHC for p62, pERK, and Ki67 of KP lung tumors from (**I**) (left panel) and quantification of pERK and Ki67 (right panel). Data are mean ± s.e.m. *P < 0.05; **P < 0.01; ***P < 0.001.

To better determine whether the combination of HCQ and Trametinib is a novel specific approach for treating KL lung tumors, we induced KL lung tumors in *Kras*^*LSL-G12D/+*^*;Lkb1*^*Flox/Flox*^ GEMMs via a single Lentiviral Cre-recombinase intranasal infection. At 12 weeks post tumor induction, when lung tumors were developed [5], mice were treated with vehicle, Trametinib, HCQ or the combination therapy for four weeks (Fig. 3A) and the tumor growth was assessed by gross lung pathology, wet lung weight (Fig. 3B), and quantification of tumor burden and tumor number of scanned lung sections with H&E staining (Fig. 3C–E). As expected, Trametinib did not inhibit the growth of KL lung tumors; however, HCQ showed a tendency to inhibit the growth of KL lung tumors. Most excitingly, the combination therapy significantly suppressed the growth of KL lung tumors compared with the vehicle control or the single agents (Fig. 3B–E). In addition, there was no significant difference in mouse body weight between the four treatment groups, indicating lack of drug toxicity (Fig. 3F). The treatment with Trametinib significantly reduced the phosphorylation of ERK, whereas HCQ treatment caused the accumulation of p62, indicating blockade of the MEK and autophagy pathways in KL lung tumors (Fig. 3G). Less KL tumor cell proliferation was observed when mice were treated with either HCQ or the combination (Fig. 3G), which is consistent with the reduced tumor growth (Fig. 3B–E). These same treatments were applied to the *Kras*^*LSL-G12D/+*^*;p53*^*Flox/Flox*^ GEMMs bearing KP lung tumors. The single agents or the combination therapy had no anti-tumor effect on established KP lung tumors (Fig. 3H–N).

### Combination of HCQ and Trametinib synergistically inhibited KL PDX tumor growth, but not KP PDX tumor growth

**Fig. 4:**
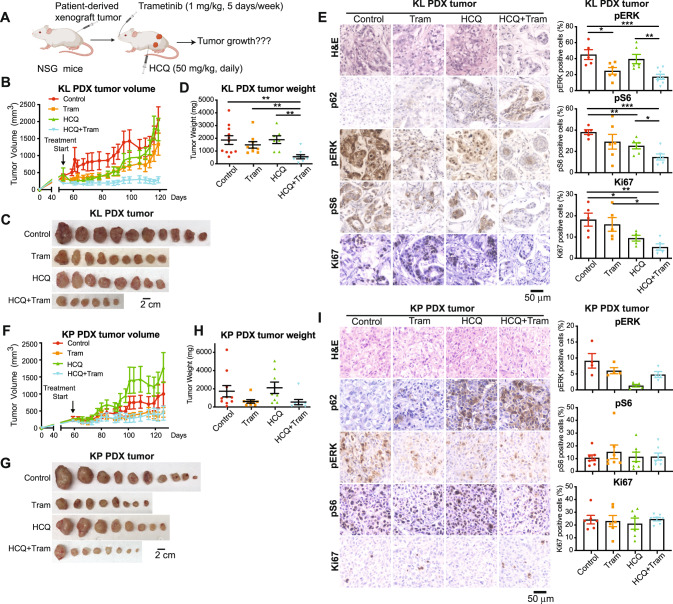
HCQ increased the sensitivity of KL PDX tumors, not KP PDX tumors, to MEK inhibitor Trametinib. **A** Scheme of Patient-Derived Xenograft (PDX) tumor model treated with vehicle control, HCQ (50 mg/kg, daily, I.P.), Trametinib (Trametinib, 1 mg/kg, 5 days/week, oral gavage), or the combination. **B** Graph of KL PDX tumor growth in NSG mice treated with vehicle control, HCQ, Trametinib or the combination for 10 weeks. **C** Gross pathology of KL PDX tumors. **D** KL PDX tumor weight at the end of experiment. **E** H&E and IHC for p62, pERK, pS6, and Ki67 (left panel) and quantification of pERK, pS6, and Ki67 (right panel) of KL PDX tumors. **F** Graph of KP PDX tumor growth in NSG mice treated with vehicle control, HCQ, Trametinib, or the combination for 9.5 weeks. **G** Gross pathology of KP PDX tumors. H. KP PDX tumor weight at the end of experiment. **I** H&E and IHC for p62, pERK, pS6, and Ki67 (left panel) and quantification of pERK, pS6, and Ki67 (right panel) of KP PDX tumors. Data are mean ± s.e.m. *P < 0.05; **P < 0.01; ***P < 0.001.

Patient-derived tumor xenograft models (PDXs) have increasingly become the preferred tool in research to translate findings for optimal treatment of the human diseases. We therefore examined the effect of combination therapy in treating PDX tumors for KL and KP NSCLC. NSG mice bearing established KL (JAX-J000095635) PDX or KP (JAX-TM00233) PDX were treated with the single agents or the combination, and tumor growth was monitored (Fig. 4A). Compared with the single agents, the combination treatment synergistically inhibited KL PDX tumor growth (Fig. 4B–D). Histology analysis found that the combination treatment increased p62 accumulation, decreased pERK and pS6, and inhibited cell proliferation (Ki67) (Fig. 4E). Although Trametinib inhibited the growth of KP PDX tumors, the addition of HCQ did not show further anti-tumor effects (Fig. 4F–I). In conclusion, through different mouse models, we demonstrated that the use of HCQ to inhibit autophagy specifically causes KL tumors, but not KP tumors, to be sensitive to the MEK inhibitor Trametinib.

### Combination of HCQ and Trametinib impaired KL lung tumor energy homeostasis

**Fig. 5:**
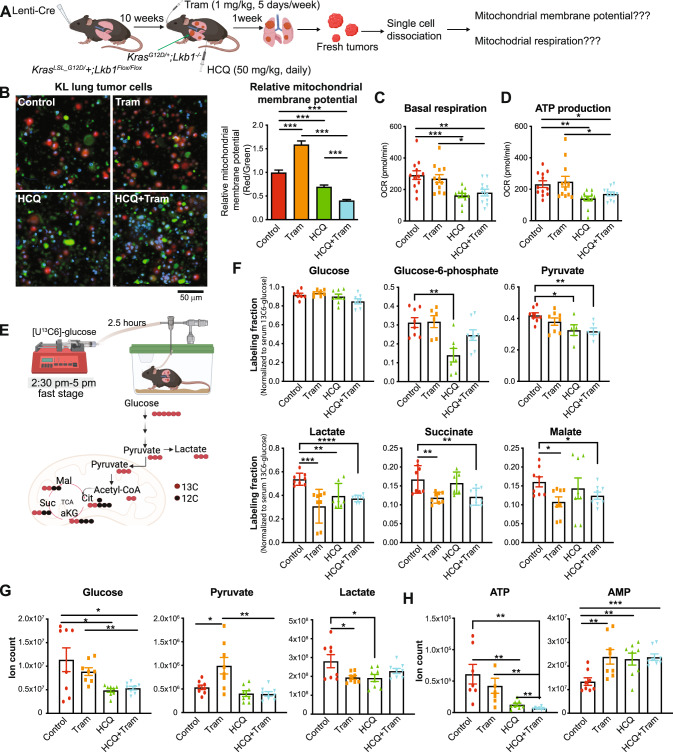
Combination of HCQ and Trametinib impaired KL lung tumor energy production. **A** Scheme to examine mitochondrial function in KL lung tumors. **B** Overlapping images of single-cell suspension from KL lung tumors of the mice treated with vehicle control, HCQ, Trametinib, or the combination for one week and stained with MitoTracker Red CMXRos for mitochondrial membrane potential and Mitotracker Green FM for mitochondrial mass (left panel); and graph of the relative mitochondrial membrane potential of single-cell suspension from KL lung tumors (the ratio of red fluorescence intensity and green fluorescence intensity) (right panel). Blue: Hoechst 33342 for nuclear staining. **C** Basal mitochondrial respiration of single-cell suspension from KL lung tumors of the mice treated with vehicle control, HCQ, Trametinib, or the combination for one week, measured by Seahorse XFe24 Analyzer. **D** ATP production of single-cell suspension from KL lung tumors of the mice treated with vehicle control, HCQ, Trametinib, or the combination for one week, measured by Seahorse XFe24 Analyzer. **E** Scheme of in vivo [U^13^C6]-glucose tracing in mice treated with vehicle control, HCQ, Trametinib, or the combination for two weeks (top panel) and ^13^C glucose carbons to glycolytic and TCA cycle intermediates (bottom panel). **F** Normalized labeling fraction of glucose and TCA cycle intermediates of KL lung tumors by infused [U^13^C6]-glucose in mice for 2.5 h. **G** Levels of glucose, pyruvate, and lactate of KL lung tumors from mice treated with vehicle control, HCQ, Trametinib, or the combination for two weeks. **H** Levels of ATP and AMP of KL lung tumors from mice treated with vehicle control, HCQ, Trametinib or the combination for two weeks. Data are mean ± s.e.m. *P < 0.05; **P < 0.01; ***P < 0.001; ****P < 0.001.

To understand the mechanism of the combination treatment in inducing KL tumor regression, we dissociated the KL lung tumors into single-cell suspension one week after treatment with the single agents or the combination and examined the mitochondrial function (Fig. 5A). We found that mitochondrial membrane potential was significantly higher in the KL lung tumor cells from mice treated with Trametinib than vehicle control mice, which was significantly reduced when combined with HCQ treatment (Fig. 5B). Compared with Trametinib alone, the combination of HCQ and Trametinib also resulted in a significant decrease in basal respiration and ATP production in KL lung tumor cells (Fig. 5C, D). Hence, as observed in the in vitro experiments (Fig. 1F–K, M), the combination therapy may impair mitochondrial function to prevent the growth of KL lung tumors in vivo.

LKB1 loss upregulates tumor glycolysis [47]. In addition, glucose is one of the major carbon sources for KL lung tumor TCA cycle intermediates [48]. To determine the impact of the combination treatment on KL lung tumor glycolysis and TCA cycle metabolism, we performed in vivo [U^13^C6]-glucose tracing and metabolic flux analysis in mice bearing KL lung tumors after two weeks of drug treatment (Fig. 5E). Total glucose enrichment was comparable in KL lung tumors of mice treated with vehicle control, HCQ, Trametinib or the combination. We observed that HCQ significantly inhibited the glucose flux to glycolytic intermediates glucose-6-phosphate, pyruvate and lactate; but had no effect on TCA cycle metabolites; the combination of HCQ and Trametinib significantly suppressed the glucose flux to glycolytic intermediates and TCA cycle intermediates (Fig. 5F). In addition, the levels of glucose and pyruvate in KL lung tumors from combination treatment were significantly lower than the tumors of mice treated with Trametinib (Fig. 5G). Moreover, compared with the vehicle control or the single agents, the ATP level of KL lung tumors in mice treated with the combination was significantly reduced (Fig. 5H). Taken together, our observations indicate that one of the mechanisms by which the combination therapy inhibits the growth of KL tumors might be the impairment of glucose-mediated metabolism, resulting in energy crisis.

### Combination of HCQ and Trametinib induced Ferroptosis in KL tumors

Ferroptosis is characterized by ROS production from accumulated lipid and iron peroxidation [49]. Autophagy promotes ferroptosis by regulating cellular iron homeostasis [50, 51]. However, many existing studies suggest that autophagy promotes drug resistance, while ferroptosis reverses drug resistance in cancer treatment [52, 53]. A recent study reported that Atg7 deletion in Cre-inducible Atg7 knockout mice decreases NRF2 levels and enhances ferroptosis in liver [54]. Increased oxidative stress is usually associated with mitochondrial injury with a marked decrease in mitochondrial membrane potential [55]. Indeed, we observed that combination of HCQ and Trametinib led to reduced mitochondrial membrane potential compared to control or Trametinib treatment alone in KL TDCLs (Fig. 1K, M). We therefore hypothesized that combination of HCQ and Trametinib may cause ferroptosis in KL tumors.

**Fig. 6:**
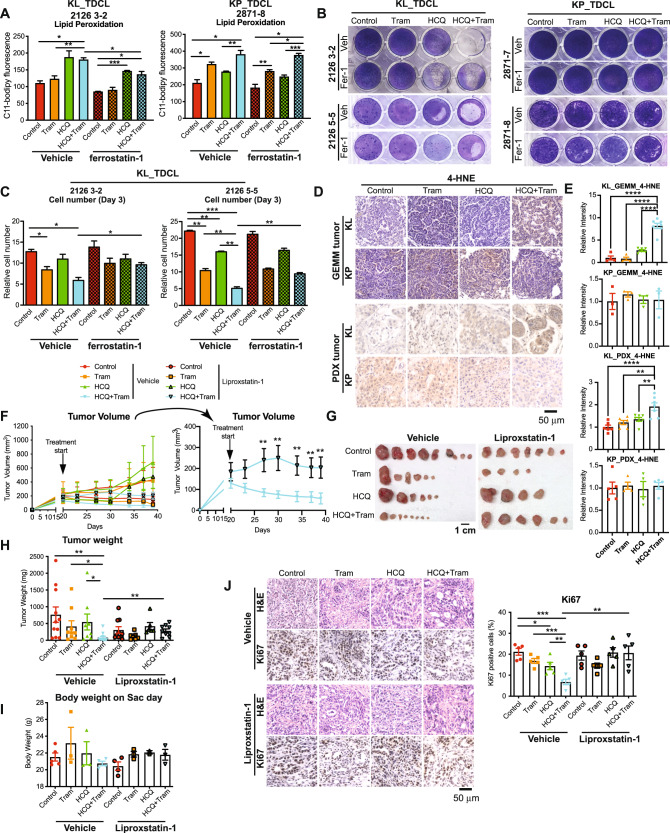
Combination of HCQ and Trametinib induced ferroptotic cell death to inhibit KL tumor growth. **A** Lipid peroxidation of KL and KP TDCLs was examined by C11-BODIPY (1 μm) staining in cells treated with vehicle control, HCQ (10 μM), Trametinib (2.5 nM) and the combination in the absence or presence of ferroptosis inhibitor ferrostatin-1 (0.5 μM). **B** Clonogenic survival assay of KL and KP TDCLs treated with vehicle control, HCQ (10 μM), Trametinib (2.5 nM) and the combination in the absence or presence of ferroptosis inhibitor ferrostatin-1 (0.5 μM). **C** Relative cell proliferation of KL TDCLs treated with vehicle control, HCQ (10 μM), Trametinib (2.5 nM) and the combination in the absence or presence of ferroptosis inhibitor ferrostatin-1 (0.5 μM). **D**, **E** IHC (**D**) and quantification (**E**) of 4-HNE staining in KL and KP lung tumors from GEMMs as well as PDX tumors. **F** Graph of KL allograft tumor growth in C57BL/6 mice treated with vehicle control, HCQ (50 mg/kg, daily, I.P.), Trametinib (1 mg/kg, 5 days/week, oral gavage), and the combination in the absence or presence of ferroptosis inhibitor Liproxstatin-1 (10 mg/kg, daily, I.P.). **G** Gross KL allograft tumor from (**F**) at the end of experiment. **H** KL allograft tumor weight from (**G**) at the end of experiment. **I** Body weight of the mice on the day of sacrifice from (**F**). **J** Representative H&E and IHC for Ki67 of KL allograft tumors from (**G**) (left panel) and quantification of Ki67 (right panel). Data are mean ± s.e.m. *P < 0.05; **P < 0.01; ***P < 0.001.

As ferroptosis is associated with lipid peroxidation [56], we first examined the lipid peroxidation in KL and KP TDCLs in the absence or presence of the ferroptosis inhibitor Ferrostatin-1 by C-11 BODIPY staining. We found that HCQ combined with Trametinib significantly increased lipid peroxidation of KL TDCLs compared with vehicle control or Trametinib alone, which was significantly inhibited by the ferroptosis inhibitor Ferrostatin-1 (Fig. 6A). In KP TDCLs, although lipid peroxidation was observed when cells were treated with Trametinib or the combination, Ferrostatin-1 treatment had no effect on lipid peroxidation (Fig. 6A). We further found that Ferrostatin-1 successfully prevented cell death and restored the reduced cell proliferation caused by the combination therapy in KL TDCLs (Fig. 6B & C).

Consistent with the in vitro data, KL lung tumors and KL PDX tumors from the mice treated with the combination therapy showed increased expression of ferroptosis marker 4-hydroxynonenal (4-HNE) compared with vehicle control and the single agents (Fig. 6D, E); this difference was not observed in KP lung tumors and PDX tumors (Fig. 6D, E). These prompted us to test the hypothesis that combination of HCQ and Trametinib may cause ferroptosis, thereby suppressing the growth of KL tumors. We therefore induced subcutaneous allograft KL tumors by injecting KL TDCLs in syngeneic C57BL/6 mice. Once the tumor size reached to 200 mm^3^, we treated the mice with the single agents or the combination in the absence or presence of the ferroptosis inhibitor Liproxstatin-1. The combination of HCQ and Trametinib significantly inhibited KL allograft tumor growth compared with the control or the single agents; while the ferroptosis inhibitor Liproxstatin-1 significantly rescued the KL allograft tumor growth in mice treated with the combination compared with vehicle control (Fig. 6F–H). There was no difference in the body weight between treatment groups (Fig. 6I), indicating lack of drug toxicities. Moreover, decreased cell proliferation was observed in the tumors of mice with combination treatment, which was abolished by the Liproxstatin-1 treatment (Fig. 6J). Taken together, we demonstrated that one of mechanisms for reducing KL tumor growth through the combination of HCQ and Trametinib might be by inducing ferroptotic cell death.

## Discussion

Autophagy promotes cancer by enhancing metabolic fitness and limiting immune and non-immune tumor suppression mechanisms to provide protection from cancer therapy [28]. This provides strong evidence that targeting the autophagy pathway with small-molecule inhibitors would be a novel therapeutic strategy. A recent preclinical mouse study and clinical trial suggest that targeting autophagy with chloroquine (CQ) increases the sensitivity of pancreatic ductal adenocarcinoma (PDAC) to MEK inhibition [29, 57]. The combination of CQ and Trametinib-induced cytotoxicity in patients with metastatic uveal melanoma [58]. Co-mutations of LKB1 or TP53 with KRAS define different subsets of NSCLC which exhibit different responses to standard cancer treatments [6, 9, 59]. Previously we found that the dependence of cell-autonomous autophagy on tumorigenesis is distinct between KL and KP lung tumors [5]. In this study, we further revealed that targeting autophagy by inhibiting lysosome function with HCQ can sensitize KL NSCLC, but not KP NSCLC, to MEK inhibitor Trametinib, suggesting that *LKB1* mutations could be explored as a predictive biomarker for precision lung cancer therapy using autophagy inhibitors.

Inhibition of the MAPK pathway using Trametinib induces autophagy [60]. Trametinib has also been shown to upregulate the expression of autophagy promoter ULK1 in human breast cancer tissues [61]. In the present study, our work suggests that autophagy acts as a resistance mechanism for KL lung tumor cells to survive MEK inhibition. Using different mouse tumor models, including allograft mouse models, GEMMs, and PDX mouse models, we provide a therapeutic strategy by combining MEK and autophagy inhibition for the treatment of LKB1-mutant KRAS-driven NSCLC. Recently, Lys05, a potent lysosomal inhibitor as well as ULK1 inhibitors ULK-101 and Vps34 inhibitors have shown promising results when used in combination with chemotherapy and immunotherapy [62–64]. In the future, it would be interesting to understand whether targeting autophagy using other autophagy inhibitors could also cause KL lung tumors to be sensitive to MEK inhibition. This can provide increased options for the treatment of KL NSCLC.

Cancer cells alter cellular metabolism to fulfill the needs of enhanced proliferation and growth [65]. Both host autophagy and tumor cell-autonomous autophagy sustain nutrients for cancer cells to survive metabolic stress [28, 29, 36, 39, 41]. Targeting autophagy by HCQ makes KL lung tumors sensitive to the MEK inhibitor Trametinib, prompting us to further elucidate the potential underlying mechanism of tumor regression caused by the combination therapy. By examining the mitochondrial membrane potential, mitochondrial oxygen consumption rate as well as in vivo [^13^C6]-glucose metabolic channel to glycolytic and TCA cycle intermediates, we found that the combination of HCQ and Trametinib impaired glucose-mediated metabolism and caused mitochondrial dysfunction, thereby resulting in the energy crisis.

Several cell death pathways have been discovered during cancer treatment. Ferroptosis is a type of controlled cell death marked by the iron-dependent aggregation of lethal amounts of lipid hydroperoxides [49]. Tumorigenesis induction of the PI3K-AKT-mTOR pathway inhibits ferroptosis through SREBP-mediated lipid synthesis; and suppression of this pathway improves the chemotherapeutic impact of ferroptosis induction [52]. Ferroptosis is, indeed, associated with severe damage in mitochondrial morphology, bioenergetics, and metabolism [66]. Increased oxidative stress is associated with mitochondrial damage and decreased mitochondrial membrane potential [66], which was observed in KL cancer cells with combination treatment. This prompted us to test the hypothesis that dysfunctional mitochondria in KL cancer cells caused by the combination treatment may trigger ferroptosis. Indeed, we observed an increased lipid peroxidation and cell death in KL TDCLs in the combination treatment, which was rescued by ferrostatin-1, a ferroptosis inhibitor. In addition, Liproxstatin-1, a ferroptosis inhibitor for in vivo study, restored KL tumor growth that was suppressed by the combination therapy. As such, our observations suggest that the combination of HCQ and Trametinib induces ferroptotic cell death, resulting in a decrease in KL allograft tumor growth. The interrelationship between autophagy and ferroptosis has recently attracted more attention. In particular, numerous studies reported that autophagy promotes drug resistance, while ferroptosis is generally considered to reverse drug resistance in cancer. Moreover, ferroptosis can be induced by autophagy upon the elimination of ferritin in the cancerous cells [50]. Like cellular metabolites including lipids, amino acids and carbohydrates can induce ferroptosis, treatment with inhibitors of MEK signaling together with autophagy inhibition causes a devastating type of oxidative stress: ferroptosis-inducing lipid peroxidation [65, 67, 68]. Our current study found that autophagy inhibition alone did not induce ferroptosis in KL tumor cells; rather, inhibition of autophagy sensitized KL tumors to MEK inhibitor by inducing ferroptotic cell death. Therefore, in the future, it will be interesting to understand how KL cancer cells “decide” to respond to Trametinib by preferentially undergoing ferroptosis or autophagy and determine how ‘ferroptotic’ and ‘autophagic’ processes work alone or synergistically to improve the prognosis of patients with cancer. In addition, it would be of great value to assess if the combination of HCQ and Trametinib can further sensitize KL lung tumors to ferroptosis inducer, which may provide a potential therapeutic strategy for the treatment of this subtype of NSCLC. Although combination therapy induces ferroptosis, other mechanisms might be involved in the reduction of KL tumor growth, which requires further investigation.

## Materials and Methods

### Cell culture

KL (2126 3-2, 2126 5-5) and KP (2871-7 and 2871-8) TDCLs were generated from mouse KL or KP lung tumors as reported previously [5, 30]. TDCLs were cultured in RPMI-1640 medium containing 10% fetal bovine plasma (FBS), 1% penicillin/streptomycin, and 0.075% sodium bicarbonate at 37 °C with 5% CO_2_.

Cell lines were routinely confirmed to be free of mycoplasma using the MycoAlert Mycoplasma Detection Kit (Lonza).

### Mice

Rutgers Animal Care and Use Committee (IACUC) has approved all animal experiments performed in this study. *Kras*^*LSL*^*-*^*G12D/+*^*;Lkb1*^*flox/flox*^ and *Kras*^*LSL_G12D/+*^*;p53*^*flox/flox*^ mice were generated in our previous study [5, 30]. Mice (equal numbers of males and females) were intranasally infected with Lentiviral-Cre (UI Viral Vector Core) at 5 × 10^6^ pfu per mouse at 6–8 weeks of age to induce KL and KP lung tumors. C57BL/6 mice were ordered from Rutgers CINJ Animal Breeding Core Facility. 6–8 weeks NCr nude mice (CrTac:NCr-Foxn1^nu^) were ordered from Taconic. NSG mice (Strain #: 005557) at 6–8 weeks were ordered from the Jackson Laboratory. JAX-J000095635 (co-mutations of KRAS and LKB1: KL_PDX) and JAX-TM00233 (co-mutations of KRAS and TP53: KP_PDX) mice were purchased from the Jackson Laboratory and PDX tumors were implanted into the left and right back flank of male NSG mice.

For allograft tumor induction, KL (2126 3-2) TDCLs and KP (2871-8) TDCLs were subcutaneously injected into the right and left flank of male NCr nude mice or male C57BL/6 mice at 2 × 10^6^ cells/injection at 6–8 week of age.

For the drug treatment of mice bearing allograft tumors or PDX tumors, when a tumor size reached 200 mm^3^, mice were randomly divided into four groups and treated with vehicle control, HCQ (50 mg/kg, intraperitoneal route (I.P.), daily), Trametinib (1 mg/kg, oral gavage, 5 days/week), or the combination. Liproxstatin (MedChemExpress, Cat#: HY-12726) was administered to a group of mice at 10 mg/kg, I.P., daily. *n* = 5 for each treatment group. Tumor volume was measured using a caliper three times a week. Treatment was continued until a humane endpoint was reached to sacrifice the mice and tumors were collected for histological analysis.

For the treatment of mice bearing KL or KP lung tumors, at 12 weeks post tumor induction, mice were randomly divided into four groups and treated with vehicle control, HCQ (50 mg/kg, I.P., daily), Trametinib (1 mg/kg, oral gavage, 5 days/week), or the combination for four weeks; subsequently, mice were sacrificed for histology analysis. *n* ≥ 5 for each treatment group.

For intravenous infusion experiments, a jugular vein catheterization surgery was performed in mice bearing KL lung tumors. *n* ≥ 3 for each treatment group. After 5 days of recovery post-catheterization, [U^13^C6]-glucose tracer was infused at 0.1 μl/g/min rate for 2.5 h (2:30–5 pm) during the fasting stage. At the end of the infusion, blood was collected to analyze circulating plasma metabolites. Mice were euthanized via cervical dislocation, tumors were collected and immediately clamped between metal plates at liquid nitrogen temperature.

For the drug treatment, tumor-bearing mice were randomly divided into each treatment group and treated blindly.

### Reagents

[U^13^C6]-glucose was purchased from Cambridge Isotope Laboratories (Cat#: CLM-1396). Hydroxychloroquine sulfate was purchased from ACROS Organics. Ferrostatin-1 (Cat#: SML0583-5MG) was purchased from Sigma Aldrich. BisBenzimide H33342 trihydrochloride (Hoechst) (Cat#: 14533) was purchased from Sigma Aldrich. MitoTracker Red CMXRos (Cat#: M7512) and MitoTracker Green FM (Cat#: M7514) were purchased from Invitrogen. TMRM Assay Kit (Mitochondrial Membrane Potential) (Cat#: ab228569) was purchased from Abcam.

### Tumor burden quantification

Hematoxylin and eosin (H&E)-stained lung specimens were imaged at Rutgers Cancer Institute of New Jersey Biomedical Informatics shared resource using an Olympus VS120 whole slide scanner (Olympus Corporation of the Americas, Center Valley, PA) at 20x magnification. The image analysis protocol was custom developed on the Visiopharm image analysis platform (Visiopharm A/S, Hoersholm, Denmark) to identify tissue area and compute tumor burden based on semi-automatically detected tumors. Tumor masks and whole-tissue masks were computed from low-resolution image maps, which were extracted from whole slide images. Tumor and whole-tissue masks were created for each slide. The segmentation masks were used for the generation of ratios of tumor burden.

### Histology and IHC

For IHC, paraffin-embedded sections were stained with antibodies against p62 (Enzo Life Sciences, PW9860-0100), Ki67 (Abcam, ab-15580), pS6 (Cell Signaling, 4858S), p42/44 MAPK (p-ERK) (Cell Signaling, 4376), and 4-hydroxynonenal (4-HNE) (Abcam, ab46545) as described previously [30].

For quantification of IHC for Ki67, pS6, p-ERK and 4-HNE, 6–10 representative images from each treatment group were obtained and scored using the ImageJ software.

### Mitochondrial membrane potential

KL and KP TDCLs were seeded at 1 × 10^4^ cells in 96-well plates overnight. The next day, cells were preincubated in RPMI supplemented with vehicle, HCQ, Trametinib or the combination treatment for 6 h. Media was aspirated and fresh media containing MitoTracker Red CMXRos (400 nM) to stain mitochondrial membrane potential and Mitotracker green (600 nM) to stain mitochondrial mass together with Hoechst 33342 (1:1000) for nuclear staining were added to the cells, incubated for 15 min and images were taken using the Nikon A1R-Si confocal microscope system.

For measuring KL and KP TDCLs mitochondrial membrane potential using TMRM (ab228569), after 6 h treatment as described above, cells were incubated with 1X Live Cell Imaging Buffer provided in the kit (ab228569) containing 200 nm TMRM to stain TMRM that localizes in the mitochondria for 30 min. The Hoechst 33342 for nuclear staining was added (1:1000) and incubated for 10 min, and images were taken using the Nikon A1R-Si confocal microscope system.

For measuring mitochondrial membrane potential in KL lung tumors, after one-week treatment with the single agents or the combination, mice were sacrificed and single-cell suspension of KL lung tumors was immediately prepared using a tissue dissociation kit (Miltenyi Biotech, 130-096-730). Subsequently, cells were resuspended in fresh media containing MitoTracker Red CMXRos (400 nM) and green (600 nM) together with Hoechst 33342, plated at a density of 2 × 10^5^ cells/well in 96-well plates, centrifuged for 1 min at 200 *g*, and incubated for 15 min. Images were taken using the Nikon A1R-Si confocal microscope system.

Fluorescence images were analyzed using the NIS Elements AR 3.2 software to calculate the mean fluorescence intensity. n ≥ 30 fields/group. Data represent the mean ± s.e.m combined from three independent experiments.

### Cell proliferation assay

Cells were seeded at 2 × 10^4^ cells per well in 12-well plates in nutrient-rich RPMI conditions. The next day, media was aspirated and cells were treated with fresh media containing vehicle, HCQ, Trametinib or their combination in the presence or absence of ferroptosis inhibitor Ferrostatin-1; subsequently, cells were trypsinized off the culture dishes and then counted in triplicates for four days using a Vi-Cell Counter.

### Cell viability assay

MTS (3-(4, 5-dimethylthiazol-2-yl)-5-(3-carboxymethoxyphenyl)-2-(4-sulfophenyl)-2H-tetrazolium) assay was performed to measure the cellular viability of KL and KP TDCLs upon treatment with HCQ, Trametinib or the combination. Briefly, cells were seeded at 7.5 × 10^3^ cells per well in 96-well plates in nutrient rich-RPMI conditions. The next day, fresh media containing vehicle, HCQ, Trametinib, or the combination at various concentrations (as listed in Table 1) were added to the wells and incubated for 72 h. MTS reagent was added as per the manufacturer’s instructions (Promega) and absorbance was measured at 490 nm. Each experimental condition was performed in quadruplicate and all experiments were conducted three times. The combination index (CI) was calculated using the Calcusyn software.

### Clonogenic survival assay

Clonogenic survival assay were performed as described previously [30, 42] and stained with 1X Giemsa (Sigma-Aldrich). Briefly, cells were seeded at 2 × 10^4^ cells per well in 12-well plates in nutrient-rich RPMI conditions. The next day, media was aspirated and cells were treated with fresh media containing vehicle, HCQ, Trametinib or their combination in the presence or absence of ferroptosis inhibitor Ferrostatin-1 for 72 h. Media was aspirated and ice-cold ethanol was added to the wells for 5 min to fix the cells. The cells were then stained with 1X Giemsa for 30 min.

### Assessment of oxygen consumption rate (OCR)

OCR of TDCLs was measured using an Agilent Seahorse XFe24 Analyzer as described previously [42, 45]. Briefly, cells were seeded at 3 × 10^4^ cells in the XF24 plates overnight prior to XF assay. Cells were preincubated in RPMI supplemented with the vehicle, HCQ, Trametinib, or the combination treatment for 6 h, and OCR was measured subsequently.

The OCR of KL lung tumors was measured by preparing single-cell suspension of the fresh lung tumors, using a tissue dissociation kit (Miltenyi Biotech, 130-096-730), from the mice treated with single agents or the combination treatment for 1-week, and attachment of cells on Seahorse plates using Cell-Tek (corning, 354240). Briefly, cells were seeded at 2 × 10^5^ cells in the XF24 plates previously coated with Cell-Tek. The cell plate was centrifuged at 200 g for 1 min, incubated for 45 min and OCR was measured subsequently.

### Metabolomic analyses by LC-MS

40:40:20 (v:v) methanol:acetonitrile:water with 0.1 M formic acid solution followed by neutralization with 15% (m:v) ammonium bicarbonate were used to extract metabolites from cells and KL lung tumors for LC-MS analysis, as previously described [45, 48].

To extract metabolites from cells, KL TDCLs were cultured in 6-cm dishes in triplicate, treated with RPMI supplemented with the vehicle, HCQ, Trametinib, or their combination for 6 h and then quickly washed twice with warm PBS; subsequently, cells were incubated with 0.7 mL of 40:40:20 methanol:acetonitrile:water with 0.5% formic acid solution on ice for 5 min followed by neutralization with 50 μL of 15% ammonium bicarbonate. The cells were then scrapped from the plates using a cell lifter, transferred to 1.5 mL freshly labeled tubes on ice and centrifuged at 4 °C for 10 min at 13,000 rpm. The supernatants were transferred to LC–MS autosampler vials (on ice) and sent for LC-MS analysis.

To extract metabolites from lung tumors, the mice bearing KL lung tumors were euthanized and lung tumors were quickly dissected and snap-frozen in liquid nitrogen with a pre-cooled Wollenberger clamp. Subsequently, frozen tissue samples were first weighed (~20–30 mg each sample) and ground using a Cryomill (Retsch, Newtown, PA) in liquid nitrogen at 25 Hz for 2 min. The resulting powder was then mixed with −20 °C 40:40:20 methanol:acetonitrile:water with 0.1 M formic acid, followed by 10 s of vortexing, 10 min incubation on ice, neutralization with 50 μL/mL of 15% ammonium bicarbonate, and 10 min centrifugation at 4 °C and 16,000 *g*. The supernatants were transferred to LC–MS autosampler vials (on ice) and sent for LC-MS analysis.

To extract metabolites from plasma, blood from the mice bearing KL lung tumors was collected using submandibular bleeding in Heparin coated tubes. The blood was centrifuged at 8000 rpm for 8 min, and plasma supernatant was aliquoted in Eppendorf tubes and stored at −80 °C. On the day of the experiment, 5 μL plasma extract was kept on ice and mixed with −20 °C 40:40:20 methanol:acetonitrile:water, followed by 10 s of vortexing, 10 min incubation on ice, and 10 min centrifugation at 4 °C and 16,000 *g*. The supernatants were transferred to LC–MS autosampler vials (on ice) and sent for LC-MS analysis.

LC − MS analysis of the metabolites was performed on the Q Exactive PLUS hybrid quadrupole-orbitrap mass spectrometer (Thermo Scientific) coupled to hydrophilic interaction chromatography (HILIC). The LC separation was performed on Vanquish Horizon UHPLC system with an XBridge BEH Amide column (150 mm × 2.1 mm, 2.5 μM particle size, Waters, Milford, MA) with the corresponding XP VanGuard Cartridge. The liquid chromatography used a gradient of solvent A (95%:5% H2O:acetonitrile with 20 mM ammonium acetate, 20 mM ammonium hydroxide, pH 9.4), and solvent B (20%:80% H2O:acetonitrile with 20 mM ammonium acetate, 20 mM ammonium hydroxide, pH 9.4). The gradient was 0 min, 100% B; 3 min, 100% B; 3.2 min, 90% B; 6.2 min, 90% B; 6.5 min, 80% B; 10.5 min, 80% B; 10.7 min, 70% B; 13.5 min, 70% B; 13.7 min, 45% B; 16 min, 45% B; 16.5 min, 100% B. The flow rate was 300 μl/min. Injection volume was 5 μL and column temperature 25 °C. The MS scans were in negative ion mode with a resolution of 70,000 at m/z 200. The automatic gain control (AGC) target was 3 × 10^6^ and the scan range was 75−1000. Metabolite features were extracted in MAVEN [69] and the isotope natural abundance was corrected using AccuCor [70].

### Western blotting

KL and KP TDCLs were treated with nutrient rich RPMI media containing vehicle, HCQ, trametinib or their combination for 8 h. Media were aspirated and the cells were rinsed with PBS twice. Cell lysates were collected using the Tris lysis buffer. Protein concentration was assessed using the Bio-Rad BCA reagent. The following antibodies were used for Western blots: LC3 (Novus Biologicals, NB600-1384), total ERK (Cell Signaling, 4695), phosphorylated ERK (Cell Signaling, 9101), total S6 (Cell Signaling, 2217), phosphorylated S6 (Cell Signaling, 4858), and β-actin (Sigma, A1978). LC3 quantification was performed using Image Lab software.

### Illustration tool

The schematic images are created with BioRender.com.

### Statistics

All in vitro cell culture experiments were repeated three times. Statistical analyses were carried out with GraphPad Prism version 9.0 or Microsoft Excel. One-way or two-way ANOVA was used with Tukey’s post-hoc test for comparisons of 3 or more groups. Student’s *t* test was used for 2 group comparisons to determine nominal significance. Data were expressed as the mean ± s.e.m. A *P* value less than 0.05 was considered statistically significant. Asterisks displayed in the figure denote statistical significance. *p < 0.05, **p < 0.01, ***p < 0.001, ****p < 0.0001.

## Supplementary information

Supplemental Figure 1

## Data Availability

All datasets generated and analyzed during this study are included in this published article and its Supplementary Information files. Additional data are available from the corresponding author on reasonable request.
