# Supplementary figures and images for: Inhibition of autophagy and MEK promotes ferroptosis in Lkb1-deficient Kras-driven lung tumors
Source: Cell Death Dis. 2023 Jan 26;14(1):61. doi: 10.1038/s41419-023-05592-8 (PMC9879981; doi:10.1038/s41419-023-05592-8)

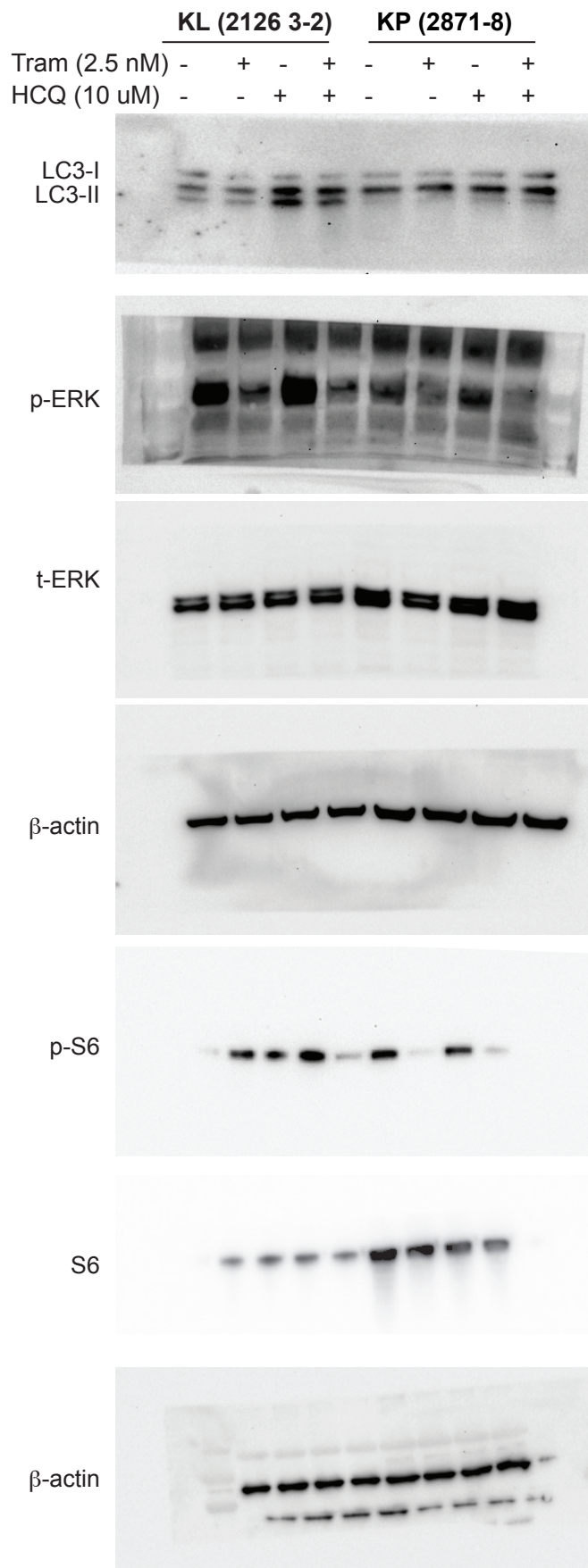

Original WB for Fig. 1E

Supplement: Supplementary file 1 — Supplemental Figure 1 [file 41419_2023_5592_MOESM1_ESM.pdf]
